# Impact of severe necrotizing fasciitis on quality of life in the Netherlands

**DOI:** 10.1007/s00068-022-02011-z

**Published:** 2022-06-09

**Authors:** Sander F. L. van Stigt, Tim K. J. Schrooten, Merel Knubben, Edward C. T. H. Tan

**Affiliations:** 1 Department of Surgery, Traumasurgery, Medical Spectrum Twente, Koningsplein 1, 7512 KZ Enschede, The Netherlands; 2 Department of Surgery, Traumasurgery, Radboud University Medical Center, Nijmegen, The Netherlands

**Keywords:** Necrotizing fasciitis, HRQoL, Quality of life, ICU

## Abstract

**Purpose:**

Necrotizing fasciitis (NF) is a severe soft-tissue infection which can leave survivors with big and multiple disfiguring alterations to their bodies, which can negatively affect the lives of patients by causing functional limitations and altered self-perception. In this study we aim to find if NF affect (self-reported) quality of life (QoL) in patients surviving NF.

**Methods:**

All patients with (histopathological or surgical confirmed) NF who were admitted to the intensive care unit for 24 h or more between January 2003 and December 2017 in five hospitals from the Nijmegen teaching region were included. Quality of life was measured with the SF-36 and WHOQol-BREF. These results were compared to reference populations from the Netherlands and a Australian reference population.

**Results:**

44 out of 60 patients (73.3%) who were contacted returned the surveys and were eligible for analysis. These patients showed lowered levels of quality of life on multiple domains of the SF-36: physical functioning, role limitations due to physical health, vitality and general health. The physical domain of the WHOQol-BREF showed also significant lowered levels of quality of life.

**Conclusion:**

NF is a severe illness with a high morbidity and mortality rate. This study shows that patients who do survive NF have decreased (self-reported) quality of life in multiple domains with a focus on decreased physical functioning. During and after admission realistic expectations should be discussed and there should be more attention to signs of permanent disability. That way extra support by a physiotherapist or social worker can be provided.

## Background

Necrotizing fasciitis (NF) is an uncommon, life threatening soft-tissue infection that progresses rapidly. It involves necrosis of the superficial fascia and subcutaneous tissue, leading to severe systemic toxicity [1]. NF is an infection that requires emergent surgical intervention, hemodynamic support on an Intensive Care Unit and targeted antibiotic treatment. In the past decades, lower mortality was achieved by improving treatment modalities, but is still described around 30% [2]. The patients who survive their infection tend to require prolonged periods of hospitalization and have, as result of radical debridement and multiple separate operations, large and often complex wounds that require soft-tissue coverage [3]. These wounds can result in disfiguring scars with defects in body contour. An amputation rate of 15.9% have been reported [4].

Changes in skin and underlying tissue can cause functional limitations, consequences for self-perception and, therefore, can negatively affect patients [5–7]. The impact of such factors has been well documented in patients surviving major burns. Many factors were found to predict lowered health related quality of life(HRQoL): severity of burns, length of hospital stay (LOS), higher age, female gender, burns to the face/hand, visible scars but also mental factors like depression afterwards, post-traumatic stress symptoms, an avoiding coping style and less emotional and social support. A variety of subdomains was affected shortly after the burn but improved over time. The most affected subdomains were: work, treatment regimen and heat sensitivity (BSHS-B questionnaire), emotional functioning (SF-36 questionnaire) and physical functioning and pain/discomfort (EQ-5D) [8–12].

Substantially less literature can be found about the quality of life in patients who survived NF. Suijker et al. reported lowered levels of HRQoL in the physical domains physical functioning, role limitations due to physical health and general health [13]. Pikturnaite et al. showed that the subdomains vitality, physical function, role limitations due to physical functioning were the lowest in their study [14]. Czymek et al. research about QoL after Fournier’s gangrene showed similar results as Suijker et al., lowered levels of HRQoL in the physical domains [15]. Urbina et al. found that patients suffering from necrotizing skin and soft-tissue infections (NSTI) had lower levels of HRQoL in multiple subdomains when compared to non-NSTI septic shock patients and when compared to NSTI patients which were not admitted to the ICU during hospitalization [16]. When compared to the research about burns these research are all much smaller (number of cases range from 10 to 30) and no systematic review has been performed yet.

Patients with NF use substantially more healthcare resource utilization (HRU) when compared with patients suffering from burns [6].

The aim of our study was to assess the quality of life of patients who have survived NF.

## Methods

### Study design

The study was designed as a retrospective cross-sectional study. Patients who were treated for NF in one of five different hospitals in the same teaching region were contacted to be included in this study. We have received a non WMO declaration from the medical ethical testing commission (METC; NW2018-06). All local feasibility committees of each of the hospitals approved this study.

### Patients

Our database contains all patients with a necrotizing fasciitis in five different hospitals between January 2003 and December 2017 [Radboud University Medical Centre Nijmegen (Radboudumc), the Elisabeth Tweesteden Hospital Tilburg (ETZ), the Gelderse Vallei Hospital Ede (GVH), Rijnstate Hospital Arnhem (RH) and Slingeland Hospital Doetinchem (SH)]. These hospitals belong to the same teaching region.

Patients with histopathological or surgical confirmed NF with a length of stay on the intensive care unit (ICU) for ≥ 24 h were included. Patients who had other forms of soft-tissue infections or a shorter or no admittance to the ICU were excluded because we only wanted to include patients with a fulminant course of NF. Patients were found through the hospitals specific patient data system using diagnostic and procedure codes.

Four patients from de ETZ hospital were asked for a focus-group in association with the department of psychology. Based on the results of the focus-group meeting we decided to use two questionnaires that are validated for measuring quality of life and the best match with this specific population of patients.

The first one is the medical outcomes short form-36 (SF-36). This is a 36-item survey with a score range of 0 to 100. The SF-36 is a well investigated and accredited suitability in the assessment of a person’s perception of changes to their health, functional limitations in performing simple daily living tasks and their ability of successfully integrating themselves into society [17].

The second was the world health organization quality of life–bref (WHOQoL-Bref). The WHOQoL-BREF instrument comprises 26 items, which measure the following broad domains: physical health, psychological health, social relationships, and environment [18]. The WHOQoL-Bref has two possible scoring ranges: one ranging van 4 to 20 and the other ranging from 0 to 100. In this research we have to use both because the used reference populations use a different scale to describe their results.

From the database we selected the patients who were eligible for the study. Taking into account the recommendations of the local review committees. We only included patients who survived NF and did not have any form of cognitive impairment before or after their period of hospitalization. All patients were contacted by telephone to obtain a verbal consent to participate in the study. We send the packages of questionnaires (demographic, SF-36 and WHOQoL-Bref) by post, together with a personalized letter, an informed consent form and a stamped return envelope.

### Statistical analysis

Results of the returned questionnaires were entered into a database created in Researchmanager™, version 5.38.0.7. Statistical analysis was performed using SPSS version 25 software (IBM, Chicago, IL, USA).

Results from our study population were compared with one Dutch reference population for the SF-36 and the two reference populations for the WHOQol-BREF questionnaires (a small Dutch reference population and a bigger Australian reference population) [19–21]. Comparison of those results was done by comparing the means of all categories and testing for statistical significance using an independent *t* test. A *p* value of < 0.05 was considered statistically significant.

## Results

### General

**Fig. 1:**
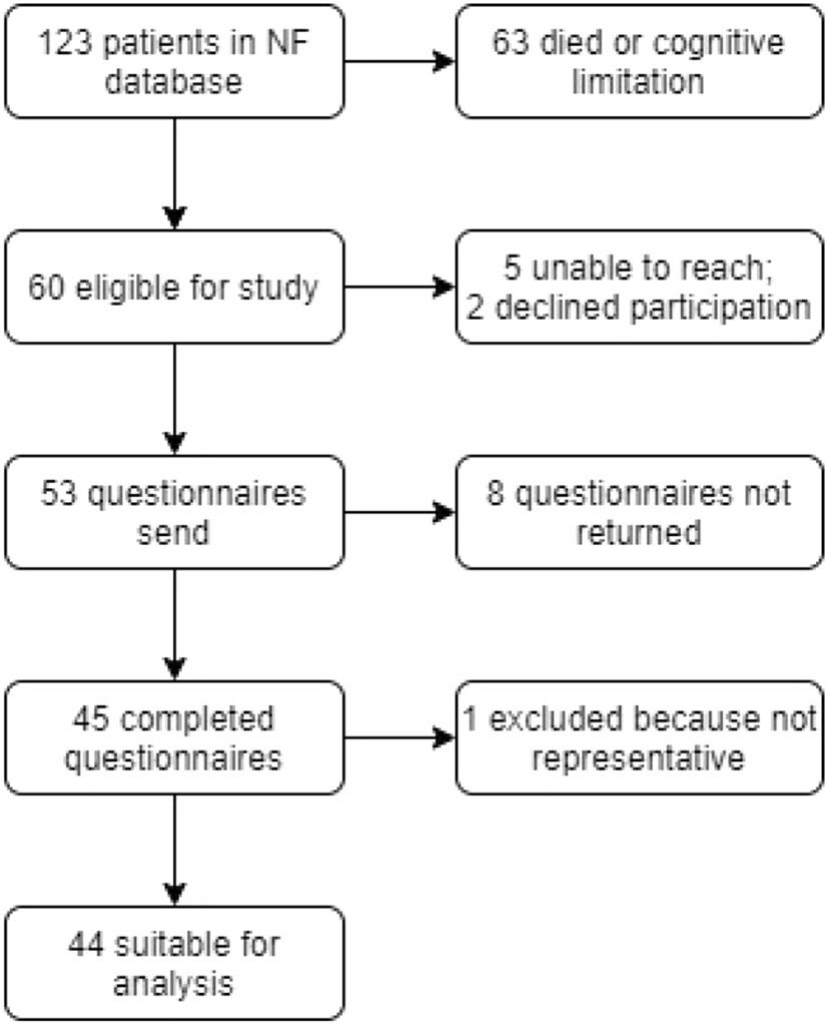
Inclusion process of patients

45 of 53 approached patients completed the survey (85%) (Fig. 1). One survey wasn’t representative, because of another very recent hospitalization (not NF). This survey was excluded for analysis. Mean follow up of the remaining 44 patients since the NF episode was 61 months (range 7–154).

Table 1 shows the demographics and characteristics of the responders compared with the survivors. Twenty-seven of the responders were male (61.4%) and seventeen were female (38.6%). Mean age was 53.6 years (range 29–76). Mean duration of hospital admission was 34.9 days (range 6–98), including a mean admission on ICU of 8.2 (range 1–30) days. The most often affected part of the body were the lower extremities (*n* = 19; 43.2%), followed by the abdomen (*n* = 12; 27.3%). The most common comorbidities were obesity (*n* = 17; 38.6%), diabetes (*n* = 11; 25.0%) and cardiovascular disease (*n* = 13; 29.5%).

**Table 1:** Characteristics survivors and responders

	Survivors in database (*n* = 84)	Responders (*n* = 44)
Age (years)	54.1	53.7
Gender (% male)	53 (63.1)	27 (61.4)
Comorbidities (%)^a^	63 (75)	32 (72.7)
Obesity	25 (29.8)	17 (38.6)
Diabetes	21 (25.0)	11 (25.0)
Immunocompromised	8 (9.5)	4 (9.1)
Kidney disease	6 (7.1)	2 (4.5)
Cardiovascular disease	28 (33.3)	13 (29.5)
Alcohol abuse	7(8.3)	2 (4.5)
Chronic liver disease	3 (3.6)	1 (2.3)
Malignancy	13 (15.5)	3 (6.8)
Affected body part (%)		
Head	6 (7.1)	4 (9.1)
Thorax	8 (9.5)	3 (6.8)
Abdomen	25 (29.8)	12 (27.3)
Upper extremity	10 (11.9)	4 (9.1)
Lower extremity	32 (38.1)	19 (43.2)
Fournier	17 (20.2)	5 (11.4)
Type NF (%)	Type 1: 48 (57.1)	Type 1: 25 (56.8)
	Type 2: 31 (36.9)	Type 2: 16 (36.4)
	Unknown: 5 (6.0)	Unknown: 3 (6.8)
Length of stay (days)		
ICU	7 (1–142)	5.5 (1–30)
Ward	22 (0–84)	21.5 (0–80)
Total	32 (2–177)	28.5 (2–98)
Number of operations (#)	3.5 (1–13)	4 (2–13)
Amputation (%)	5 (6.0)	3 (6.8)
VAC (%)	50 (59.5)	28 (63.6)
SSG (%)	41 (48.8)	23 (52.3)

Continuous data is presented as mean with standard deviation and range

*ICU* intensive care unit, *VAC* vacuum assisted closure, *SSG* split skin graft

^a^Number of diagnosis that patient had prior to presentation with NF: obesity, diabetes, cardiovascular history, malignancy, immune compromised, renal insufficiency, alcohol abuse, chronic liver disease, drug use or other

Aside from the SF-36 and WHOQoL-BREF questionnaires respondents filled in a questionnaire with questions regarding their demographics. Thirty-four patients were in a relationship, 10 were single. Sixteen of the respondents were retired at the time of filling in the questionnaires. Eight of the remaining 28 patients (28.6%) were incapacitated for work. Time to return to work after the diagnoses of NF was 7.7 months (range 2–24).

### Results of the SF-36

When compared to a Dutch reference population, the study population showed significant decreases in four self-reported categories [19]. In the studied population self-reported physical functioning scored 62.8 (± 26.5). This is significantly lower than the Dutch reference population which scored 83.0 (± 22.8) (*p *< 0.0001). Role limitations due to physical health scored 60.8 (± 42.9) in the study population and 76.4 (± 36.3) in the reference population, which is significantly lower as well (*p* = 0.0051). Vitality was lower in the studied population 59.6 (± 19.6) vs. 68.6 (± 19.3) in the reference population (*p* = 0.0022). Lastly, general health was found to be significantly lower also, 58.1 (± 24.4) vs. 70.7 (± 20.7) (*p *≤ 0.0001). In the other categories (emotional, role limitations due to emotional problems, social functioning and pain) no difference was found. (Table 2).

**Table 2:** Results of the SF-36 and comparison to the reference population

Category	Study population (*n* = 44) Mean (SD)	Reference population (*n* = 1742) Mean (SD)	*p* value
Physical functioning	62.8 (26.5)	83.0 (22.8)	** < 0.0001**
Role limitations due to physical health	60.8 (42.9)	76.4 (36.3)	**0.0051**
Emotional	77.4 (15.5)	76.8 (17.4)	0.8326
Role limitations due to emotional problems	82.6 (34.8)	82.3 (32.9)	0.9556
Vitality	59.6 (19.6)	68.6 (19.3)	**0.0022**
Social functioning	80.7 (19.0)	84.0 (22.4)	0.33
Pain	69.9 (23.1)	74.9 (23.4)	0.165
General health	58.1 (24.4)	70.7 (20.7)	** < 0.0001**

Significant values are in Bold (*p* <0.05)

### Results of the WHOQol-Bref

For comparison of the results of the WHOQoL-BREF two reference populations were used. A smaller Dutch reference population which was part of a bigger international research done by the WHOQOL group [20]. The second population is a bigger population from a study in Australia which sampled community residents [21]. Two separate calculations were done because the two references populations were both presented on different scales of the WHOQoL-BREF, the 4–20 and the 0–100 scale.

When comparing the study population to the Dutch reference population one category showed a significant difference. Physical score in the study population was 14.1 (± 2.8). The score in the reference population was 18.3 (± 3.0) (*p* ≤0.0001). The other categories (psychological, social and environment) showed no difference that was significant (Table 3).

**Table 3:** Results of the WHOQoL-Bref and comparison to the Dutch reference population

Category	Study population (*n* = 44) Mean SD	Dutch reference population (*n* = 41) Mean SD	*p* value
Physical	14.1 (2.8)	18.3 (3.0)	** < 0.0001**
Psychological	15.5 (2.3)	16.6 (2.8)	0.0505
Social	15.0 (2.7)	15.8 (3.3)	0.2234
Environment	15.4 (2.8)	15.9 (2.8)	0.4131

Significant values are in Bold (*p* <0.05)

Similar results were found when comparison was done with the bigger Australian reference population. Physical score was significantly lower: 62.9 (± 17.8) vs. 73.5 (± 18.1) (*p* = 0.0002). The other categories (psychological, social and environment) showed no difference that was significant also (Table 4).

**Table 4:** Results of the WHOQoL-Bref and comparison the bigger Australian reference population

Category	Study population (*n* = 44) Mean SD	Hawthorne reference population (*n* = 866) Mean SD	*p* value
Physical	62.9 (17.8)	73.5 (18.1)	**0.0002**
Psychological	70.4 (14.6)	70.6 (14.0)	0.9265
Social	68.6 (17.0)	71.5 (18.2)	0.3013
Environment	71.4 (17.7)	75.1 (13)	0.0713

Significant values are in Bold (*p* <0.05)

## Discussion

This study showed that patients who survived NF have a lower self-reported QoL for multiple domains when compared to multiple reference populations. The SF-36 domains that were decreased are: physical functioning, role limitations due to physical health, vitality and general health. For the WHOQol-Bref only one domain was decreased, the physical domain. In the SF-36 the domain physical functioning covers how activities of daily living are performed covering for example self-care and transportation. Likewise role limitations due to physical health describes difficulties in work or activities of daily living due to physical health problems. Vitality covers (feelings of) loss of energy or fatigue. Finally general health describes how patients feel about their own health. Some questions in this domain compare the subject to other people. In the WHOQoL-Bref the physical domain describes, among other things, activities of daily living, mobility, pain, energy and work capacity.

Unfortunately it was not possible to test for normality in the reference populations and aside from this only the means, standard deviations and groups sizes were available. It was not possible to use a nonparametric test because of this. We chose to use an unpaired *t* test instead. Torrance et al. have shown that using a parametric or nonparametric test did not change the results of statistical tests when testing SF-36 data [22]. Unfortunately this has not been found for when testing WHOQoL-Bref results.

The study population was compared to one Dutch reference population for the SF-36 and two reference populations for the WHOQoL-Bref, a small Dutch population and a bigger Australian population. The reason we chose to use two reference populations for the WHOQoL-Bref is the small size of the Dutch population. We used two reference populations to check for the effect of having a smaller population (and thus having less statistical power) on the results of the *t* test.

Reference populations showed different mean domain scores for different age groups and gender. The SF-36 reference population showed a significant decrease of mean scores when age is higher and for the female gender for all domains. The used reference population of the Australian WHOQoL-Bref did not test for these differences. When looking at the mean scores a decrease in mean domain score for the physical domain can be seen for higher age. The other domains showed no clear difference when comparing age and gender groups.

Our study population has a higher percentage of males when compared to the SF-36 and Australian WHOQoL-Bref population, 61% vs. 56% vs. 44% respectively (*p* = 0.0233 and *p* = 0.0345). Mean ages differ as well, 54 vs. 48 vs. 48 (*p* = 0.4811 and *p* = 0.0211). This would mean that if there is a bias when comparing these populations, we should have found a higher domain score in our study population, especially for the physical domains. However, the opposite is the case with lowered domains scores. So if there would be a bias because of the found difference in age and gender the decrease in domain scores could be even bigger.

To our knowledge, this study is the biggest study reporting on quality of life in patients who suffered from necrotizing fasciitis. Other studies (with numbers of cases ranging from 10 to 30) reporting on QoL after NF reported lower scores on physical functioning and general health [13, 15]. This study showed the same finding but an additional significantly lowered score was found for the vitality domain. Another study performed by Pikturnaite et al. found that the domains vitality, physical function, role limitations due to physical functioning were the lowest in their study [14]. However this was not tested by comparing it to a reference population. When looking at the reference population we used, vitality and general health are already the lowest scoring domains. It is difficult to conclude that this outcome is because of NF without statistical testing.

Our found sub-domain scores of the SF-36 are comparable to the results found by Suijker et al. and Pikturnaite et al. (Cyzmek et al. did not report mean scores in their article). On the physical domain we found a score of 62.8. Suijker et al. and Pikturnaite et al. found scores of 73.7 and 58.5, respectively. Role limitations due to physical health scored 60.8 in our research vs. 58.1 and 62.5 in the other two articles. General health was also similar when compared to Suijker et al., 58.1 in this study population vs. 55.0. Urbina et al. did not report exact mean scores in their article but presented them in a figure. When comparing our results to theirs al subdomains had lower mean scores except for bodily pain, which had in higher score in their research.

When setting up this study, four patients from one of the participating hospitals (ETZ) were asked to participate in a focus-group. In association with the department of psychology meetings were held to discuss in how QoL should be measured. Because of this the choice was made to use two questionnaires instead of one as mentioned earlier. We think that in this way we decreased researcher bias when choosing the correct instrument to measure QoL. 44 out of the 60 patients who were discharged alive and did not have a cognitive limitations were contacted and returned both questionnaires. Giving a response rate of 73.3%.

When comparing the research data from burns vs NF it seems that burns effect a wider spectrum of QoL subdomains. Which could mean that burns are more severe than NF. The higher amount of HRU usage suggest otherwise [6]. The found difference in lowered subdomains could also be explained by the big difference in research-size and the amount of research that is done about burns vs. NF. Another explanation for the lowered scores on the emotional/psychological scale could be difference in mechanisms of injury between burns and NF.

The criteria for inclusion of the used database was a minimal duration of ICU-admittance of 24 h. Because of this, the database consist of patients with a more fulminant course of NF excluding less severe cases. This ensures that there is no dilution by the less severe cases and the measured change in QoL is more representative of the impact that NF can have on a patient’s life. This could be named as a limitation to this study. We think that it guarantees that the found decrease in QoL is not diluted by the less severe cases of NF, where nearly no debridement was necessary. The other studies conducted about QoL after NF did not have a severity inclusion norm. Another limitation of this study is that all cases were single time measurements. We think the best alternative using a reference population from a population which is comparable to the study population.

## Conclusion

Patients who survive necrotizing fasciitis have decreased (self-reported) quality of life when measured with the SF-36 and WHOQol-BREF questionnaires. The domains in which scores were lowered are mainly the ones covering physical functioning. Our advice is to have this found difference in mind when treating these patients in hospital and during outpatient clinic appointments. Provide patients with enough information during admission to ensure more realistic expectation about their recovery. Involve physiotherapist and social workers during admission and be alert during appointments in the outpatient clinic so that patients get the help they need after discharge. This way we can ensure that everything is done to minimize the residual complaints after NF.

## Data Availability

The dataset used and/or analyzed are available from the corresponding author on reasonable request.

## Abbreviations

NF: Necrotizing fasciitis
QoL: Quality of life
HRQoL: Health related quality of life
HRU: Healthcare Resource Utilization
NSTI: Necrotizing skin and soft-tissue infections
RadboudUMC: Radboud University Medical Center Nijmegen
ETZ: Elisabeth Tweesteden Hospital Tilburg
GVH: Gelderse Vallei Hospital
RH: Rijnstate Hospital Arnhem
SH: Slingeland Hospital Doetinchem
ICU: Intensive care unit
METC: Medical ethical testing committee
SF-36: The medical outcomes short form 36
WHOQol-BREF: The World Health Organization quality of life–BREF
